# Quetiapine and Buspirone Both Elevate Cortical Levels of Noradrenaline and Dopamine *In vivo*, but Do Not have Synergistic Effects

**DOI:** 10.3389/fpsyt.2012.00082

**Published:** 2012-09-14

**Authors:** P. H. Silverstone, M. D. Lalies, A. L. Hudson

**Affiliations:** ^1^Department of Psychiatry, University of AlbertaEdmonton, AB, Canada; ^2^Department of Neuroscience, University of AlbertaEdmonton, AB, Canada; ^3^Department of Pharmacology, University of AlbertaEdmonton, AB, Canada

**Keywords:** quetiapine, buspirone, noradrenaline, dopamine, cognitive, schizophrenia, 5HT1A, microdialysis

## Abstract

Decreased cognitive ability is a significant problem in schizophrenia, and it has been proposed that augmentation of antipsychotics with 5HT_1A_ receptor agonists may improve cognitive performance. Clinical studies have been mixed but there have been no studies specifically examining the effects of combining the atypical antipsychotic quetiapine with the 5HT_1A_ receptor partial agonist, buspirone on monoamine release. This is of interest given previous evidence that monoamine release can alter cognition in schizophrenia. In the present study we measured *in vivo* levels of monoamines in the frontal cortex of Sprague Dawley rats and examined if buspirone (2.5 mg/kg i.p.), altered monoamine release both when given alone and when combined with quetiapine (10 mg/kg i.p.). We found that serotonin levels were not altered by either drug, either alone or in combination. In contrast, both buspirone and quetiapine monotherapy significantly increased release of noradrenaline (112 and 160% respectively) and dopamine (169 and 191% respectively) compared to controls. However, there were no additional increases in *in vivo* monoamine release when the combination of these drugs were given. One possible explanation for these negative findings could be that the intrinsic 5HT_1A_ agonist activity of quetiapine on its own is of such significance that it is not further enhanced by buspirone. These findings do not support clinical studies combining buspirone and quetiapine, if these were to be used on the basis of enhanced monoamine neurotransmission. These findings may also have implications for the atypical antipsychotic drugs in development which combine dopamine D_2_ antagonism with 5HT_1A_ partial agonism.

## Introduction

In schizophrenia clinically significant deficits in cognitive functioning occur frequently, with meta-analyses suggesting that cognitive functioning is reduced by between 0.5 and 1.5 SD below normal levels (Aleman et al., 1999; Johnson-Selfridge and Zalewski, 2001; Lee and Park, 2005). Treatment with atypical antipsychotics can alleviate this to some degree (Meltzer and McGurk, 1999; McGurk et al., 2005). It is likely that some atypical antipsychotics improve cognition more than others, with quetiapine being among the most effective (Riedel et al., 2007, 2010; Guo et al., 2011). Drugs such as asenapine are hypothesized to improve cognitive performance in primate studies due to effects on serotonin and dopamine turnover (Elsworth et al., 2012).

Another suggestion for the cognitive improvement seen in patients following administration of atypical antipsychotics is that it may be due to agonist actions at the 5HT_1A_ receptor (Meltzer and Sumiyoshi, 2008; Depoortère et al., 2010), possibly by addressing an underlying decrease in 5HT_1A_ receptor stimulation (Sumiyoshi et al., 1996). This suggestion is supported by findings that stimulation of 5HT_1A_ receptors results in an increase in extracellular dopamine release in the prefrontal cortex (Yatham et al., 2005). Interestingly, quetiapine is a partial agonist at the 5HT_1A_ receptor in prefrontal cortex, while having minimal affinity for these receptors in the nucleus accumbens (Ichikawa et al., 2002; Yatham et al., 2005).

To further examine this hypothesis, studies have examined the effects on cognitive performance in schizophrenic patients of adding 5HT_1A_ receptor partial agonists to antipsychotics. The first study examined the effects of adding tandospirone, a 5HT_1A_ partial agonist to ongoing treatment with typical antipsychotic drugs, and reported some improvements in cognitive functioning (Sumiyoshi et al., 2000, 2001). In another study by the same group some modest cognitive benefits were found in one study combining the 5HT_1A_ receptor partial agonist, buspirone, with atypical antipsychotics (Sumiyoshi et al., 2007), but not in another smaller study by another group (Piškulic et al., 2009). A more recent placebo-controlled study in 46 schizophrenic patients found that addition of buspirone significantly improved negative symptoms over an 8-week study period (Ghaleiha et al., 2010). Thus, there appears some clinical data to warrant further exploration of the clinical benefits of adding 5HT_1A_ receptor partial agonist, buspirone, to atypical antipsychotics. Nonetheless, there appears little understanding of possible mechanisms of such a clinical benefit. Furthermore, to date there have been no studies which have specifically focused upon the combination of buspirone with quetiapine. This may appear a logical choice for a study given the positive benefit of quetiapine on cognitive functioning (Riedel et al., 2007, 2010; Guo et al., 2011).

It is conceivable that any cognitive benefits arising from the addition of 5HT_1A_ receptor agonists are mediated via alterations in monoamine release. Supporting this suggestion is increasing evidence from animal models of cognitive changes in schizophrenia, which find alterations in monoamine release (McLean et al., 2009; Depoortère et al., 2010; Meltzer et al., 2011; Elsworth et al., 2012). Buspirone has also been shown in *in vivo* animal studies to alter monoamine release, where it has been found to increase the release of both noradrenaline (NA; Dalley et al., 1996; Gobert et al., 1999) and dopamine (Gobert et al., 1999). Therefore, we wished to determine if a combination of buspirone and quetiapine led to any synergistic increases in the release of any of these monoamines. If so, these findings might, in part, provide the rationale for a clinical trial of this combination and, in part, help explain some of the positive benefits seen in other clinical studies.

## Materials and Methods

All procedures on animals were approved by the University of Alberta Health Sciences Animal Policy and Welfare Committee, protocol number 456/07/07D and conducted according to CCAC guidelines. The methods are very similar to those we have used in other microdialysis studies (Hudson et al., 2012).

### Animals and laboratory

The experiments were performed using male Sprague Dawley rats (final weight, 270–300 g, obtained from Bioscience, University of Alberta, Canada). Animals were held in a temperature – controlled environment (23°C) on a 12-h light/dark cycle with free access to food and water. Animals were acclimatized to their environment for 7 days prior to surgery.

### Drugs

Buspirone hydrochloride (2.5 mg/kg i.p.) and quetiapine fumarate (10 mg/kg i.p.) were purchased from commercial sources (Sigma).

### Stereotaxic surgery

Rats were anesthetized with chloral hydrate (400 mg/kg i.p. Sigma) and placed in a stereotaxic frame. An incision was made in the scalp to expose the cranium and a small 1 mm hole was bored through the skull. The dura was broken and microdialysis probes were located in frontal cortex (1.2 mm lateral, 2.7 mm anterior relative to bregma and to a depth of 5.0 mm relative to dura) using a brain atlas for coordinates. Animals remained under anesthesia for the duration of the experiment with additional chloral hydrate administered as required. Core body temperature of each rat was maintained at 37°C with a homeothermic blanket and rectal thermometer (Harvard, UK). Procedures were approved by Health Sciences Animal Policy and Welfare Committee and conformed to the guidelines set by the Canadian Council on Animal Care for ethical experiments on laboratory animals.

### Microdialysis: The probes and high performance liquid chromatography

Concentric dialysis probes were constructed in-house, using stainless steel tubing (23 gage) with a 4 mm polyacrylonitrile membrane length (ID: 0.2 mm; OD: 0.3 mm) and cut off MW 40,000 probes with a silica tubing outlet (OD: 0.17 mm). Once implanted probes were perfused with artificial cerebrospinal fluid containing: 147 mM NaCl, 1 mM MgCl_2_, 1.3 mM CaCl_2_, and 3 mM KCl, at a flow rate of 2 μL/min using a syringe infusion pump (Harvard Apparatus). Dialyzate samples were collected at 20 min intervals in and immediately subject to analysis for monoamine content. Extracellular monoamine levels were taken to be stable when three consecutive samples values did not differ by 5% of one another. The content of monoamines in dialyzate samples were quantified using two independent high performance liquid chromatography (HPLC) systems (one system for NA and one for DA and 5-HT), coupled with electrochemical detection (ECD), optimized to detect monoamines and their metabolites separately. Briefly, for NA assay mobile phase A containing 2 g/L sodium acetate, 3.1 g/L citric acid, 500 mg/L octanesulfonic acid, and 70 mg/L ethylenediaminetetraacetic acid was dissolved in deionized water to which methanol (13% v/v) was added (adjusted to pH 5.0 with 10 M NaOH). Mobile phase A was delivered at a flow rate of 1.1 ml/min onto a reverse analytical column (Hichrom, ODS 3 μm of 12.5 cm length ad 4.6 mm i.d.). Extracellular NA concentration was detected using a dual 5014A electrode cell paired to a Coulochem II (ESA) electrochemical detector. First electrode potential was set at −100 mV whilst the second electrode was maintained at +250 mV. The retention time of NA was 5.5 min. For the DA and 5-HT assay mobile phase B comprised of 10.92 g/L sodium dihydrogen orthophosphate and 270 mg/L octanesulfonic acid dissolved in deionized water to which methanol (14% v/v) was added. Mobile phase B was delivered at a flow rate of 1.0 ml/min onto a reverse phase analytical column (Beckman, ODS 3 μm of 7.5 cm length and 4.6 mm i.d.). Extracellular levels of DA and 5-HT were similarly measured; the second electrode potential was set at +300 mV. The retention times of DA and 5-HT were 5.75 and 17 min respectively. The concentrations of the monoamines in a given sample were determined by interpolating the peak height obtained from standard curves.

Following implantation of dialysis probes stable baseline measures of the monoamines were established and then drugs or vehicle injected (volume 1 ml/kg) at time zero. The doses of drugs were chosen as those previously shown not to cause a maximal effect on extracellular monoamines. The effects of the treatments were monitored for 3 h and the animals euthanized by i.p. injection of euthanyl (pentobarbitone 240 mg/ml; Vetoquinol, Canada). Brains were then removed, frozen, sectioned and probe placement confirmed and only those animals that had correct placement of the probes were included in the study.

### Statistical analysis

Data were analyzed by one-way repeated measures ANOVA followed by Dunnett’s multiple comparison test for *post hoc* determination of significant differences from saline vehicle animals following drug administration, then analyzed for significant differences between treatment groups using Bonferroni’s test. This was done to allow appropriate testing for each part of the study as Bonferroni’s test is used to compare selected pairs of results (which have been pre-defined) while Dunnett’s is used to compare all results to a control result. The level of significance was set at *p* < 0.05. The results are reported as percentage of baseline.

## Results

### Effects on serotonin release

**Figure 1:**
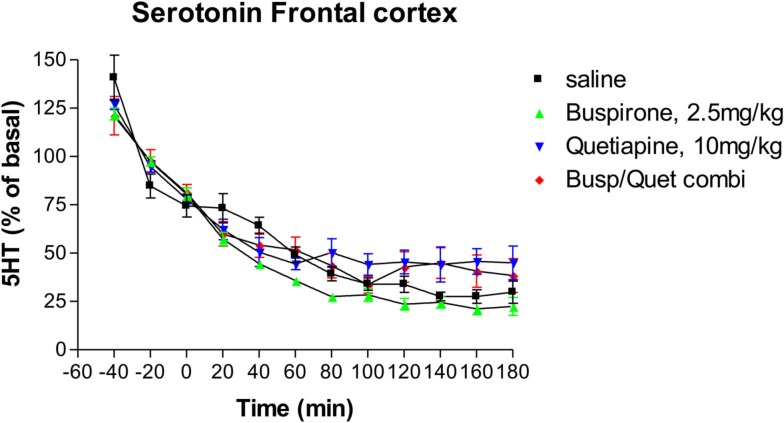
**The effect of Buspirone (2.5 mg/kg i.p.) alone (*n* = 7), Quetiapine (10 mg/kg i.p.) alone (*n* = 7), the combination of these two drugs (Busp/Quetcombi; *n* = 7), and saline vehicle control (saline; *n* = 9) on extracellular levels ofof serotonin in rat frontal cortex**. Drugs were administered at time zero, values are means ± SEM. There were no statistically significant differences between any of the four groups.

Neither buspirone alone nor quetiapine alone had any significant effect on extracellular serotonin levels (Figure 1) compared to saline controls. As with previous studies (Hudson et al., 2012), serotonin levels decreased over time in control groups. Administration of the combination of buspirone and quetiapine did not significantly alter serotonin levels compared to either saline treated controls or to either drug given alone (Figure 1).

### Effects on noradrenaline release

**Figure 2:**
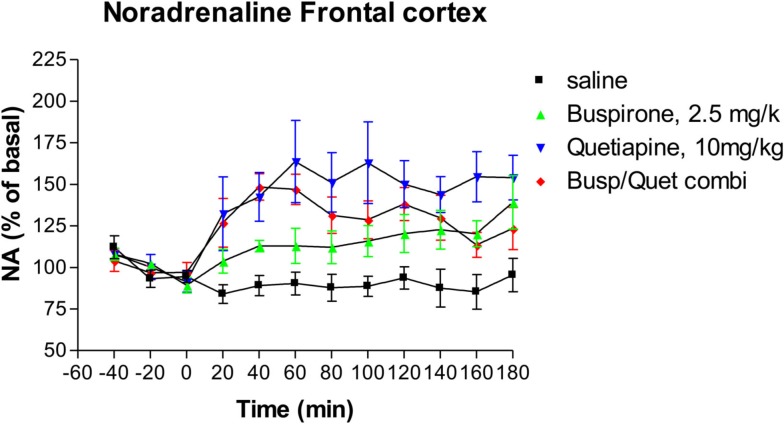
**The effect of Buspirone (2.5 mg/kg i.p.) alone (*n* = 7), Quetiapine (10 mg/kg i.p.) alone (*n* = 7), the combination of these two drugs (Busp/Quetcombi; *n* = 7), and saline vehicle control (saline; *n* = 9) on extracellular levels of noradrenaline in rat frontal cortex**. Drugs were administered at time zero, values are means ± sem. Both the buspirone alone and quetiapine alone groups were statistically different from saline controls. However, there were no statistically significantly differences between the combination of buspirone and quetiapine from either drug when given alone.

Following administration of buspirone alone there was a significant elevation of extracellular NA relative to control animals from 80 min onward (Dunnett’s Multiple Comparison Test 21.5, *p* < 0.05), as shown in Figure 2. At 180 min the mean level of NA release was 139% of baseline compared to 95% of baseline for the saline controls. Administration of quetiapine alone resulted in a significant elevation of NA compared to saline controls (Dunnett’s Multiple Comparison Test 46.1, *p* < 0.001; Figure 2). Quetiapine alone also led to significantly greater NA levels than buspirone alone (Dunnett’s Multiple Comparison Test 24.6, *p* < 0.05). The combination of buspirone and quetiapine did not significantly alter levels compared to either buspirone alone (Bonferroni’s Multiple Comparison Test 10.5, *p* > 0.05) or quetiapine alone (Bonferroni’s Multiple Comparison Test 14.1, *p* > 0.05).

### Effects on dopamine release

**Figure 3:**
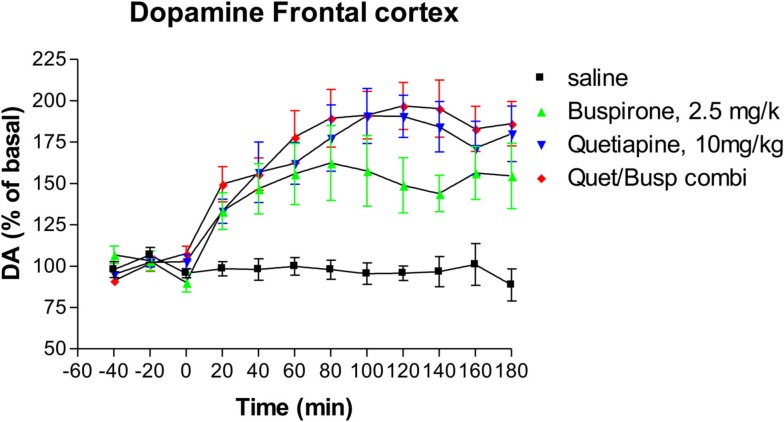
**The effect of Buspirone (2.5 mg/kg i.p.) alone (*n* = 7), Quetiapine (10 mg/kg i.p.) alone (*n* = 7), the combination of these two drugs (Busp/Quetcombi; *n* = 7), and saline vehicle control (saline; *n* = 9) on extracellular levels of dopamine in rat frontal cortex**. Drugs were administered at time zero, values are means ± sem. Both the buspirone alone and quetiapine alone group were statistically different from saline controls. However, there were no statistically significantly differences between the combination of buspirone and quetiapine from either drug when given alone.

Administration of buspirone alone caused a significant increase in the levels of extracellular dopamine (Dunnett’s Multiple Comparison Test 47.25, *p* < 0.001), an effect that lasted until the end of the experiment (Figure 3) at which point dopamine levels were 164% of baseline compared to 87% of baseline in animals who received saline. Administration of quetiapine resulted in a somewhat higher elevation of dopamine levels which was also highly significant compared to saline controls (Dunnett’s Multiple Comparison Test 56.2, *p* < 0.001), an effect that also persisted until the end of the experiment where dopamine levels were 180% of baseline (Figure 3). However, there were no statistically significant differences between the buspirone group and the quetiapine group (Bonferroni’s Multiple Comparison Test 8.94, *p* > 0.05). The combination of buspirone and quetiapine did not significantly alter levels compared to either buspirone alone (Bonferroni’s Multiple Comparison Test 15.5, *p* > 0.05) or quetiapine alone (Bonferroni’s Multiple Comparison Test 6.6, *p* > 0.05).

## Discussion

The findings from the present study found that neither buspirone or quetiapine, either alone or in combination, increased serotonin release. These findings are consistent with previous studies, using doses very similar to those examined previously (Routledge et al., 1993; Gobert et al., 1999; Yamamura et al., 2009).

In the present study we found that buspirone monotherapy increased NA release in rat frontal cortex. This is consistent with several previous *in vivo* studies which have found that buspirone reproducibly increases NA release (Dalley et al., 1996; Gobert et al., 1999), as well as reversing the locus coeruleus inhibitory effects of clonidine (Astier et al., 2003). It has been suggested that this increase is due the active metabolite of buspirone, 1-(2-pyrimidinyl-piperazine) which acts as an a_2_-adrenoceptor antagonist (Gobert et al., 1999). Additionally, buspirone increased dopamine release, which has also been reported in previous studies (Gobert et al., 1999).

In the present study we found that quetiapine significantly increased the release of both NA and dopamine, which are again consistent with the results from previous studies (Pira et al., 2004; Yamamura et al., 2009).

There have been no previous studies, however, which have examined the effects of a combination of buspirone and quetiapine on monoamine levels. The findings from the present study found no increase in the levels of any monoamine when these drugs were combined. Interestingly, there has been one small 6-week placebo-controlled study (*n* = 18) which examined the potential benefits of augmenting a variety of atypical antipsychotics with buspirone compared to placebo (Piškulic et al., 2009). For the study as a whole there were no clinical effects of the addition of buspirone on cognitive functioning compared to the placebo group. Of the 18 study patients, 4 were on quetiapine, all of whom were randomized to receive buspirone and not placebo. However, the results for this subgroup were not reported so it cannot be certain that this small subgroup did not have a different response. We are not aware of any other clinical studies of this combination.

One possible explanation for the negative results would be that both buspirone and quetiapine are acting via similar mechanisms, at least in part, to increase the release of both NA and dopamine. Supporting this proposal are findings that quetiapine is itself a partial agonist on 5HT_1A_ receptors (Pisu et al., 2010). Such a finding would also be compatible with the theory that 5HT_1A_ receptor partial agonism is an important contributor to improve cognitive performance (Meltzer and Huang, 2008). This hypothesis is likely to be examined in more detail since there are a number of novel atypical antipsychotic drugs in development which combine dopamine D_2_ antagonism with 5HT_1A_ partial agonism (Sumiyoshi et al., 2008; Newman-Tancredi, 2010; Meltzer et al., 2011). It will be of interest to see if these drugs offer enhanced effects on cognition compared to existing antipsychotics.

Should the results of the present study have been positive two further sets of research would have been justified. The first would have been to examine other antipsychotics in this model, particularly those which did not have activities at the 5HT_1A_ receptor. This would have clarified the potential role of this in the release of monoamines. Secondly, it would have been important to examine potential behavioral changes in non-anesthetized animals, particularly focusing on models that allow some possible measurement of cognitive performance. Human volunteer studies of the combination of these drugs on cognitive performance may also have been warranted. However, given the negative findings from the present study these additional studies were not carried out.

In conclusion, this study found no evidence for a synergistic effect for buspirone and quetiapine on monoamine release. It is possible these findings may be due to intrinsic 5HT_1A_ receptor partial agonism by quetiapine. However, the results of the present study do not support clinical studies in which these drugs are combined. These results also imply that any cognitive benefits that occur clinically from combining atypical antipsychotics and buspirone may not be due to increased release of monoamines.

## Conflict of Interest Statement

This study was funded in part by a grant from Biovail Corporation, to A. L. Hudson.
